# West Country Physicians Club

**Published:** 1983-04

**Authors:** 


### The West Country Physicians' Club

The Seventy-first meeting of the West Country Physicians' Club was held on the 5th and 6th November 1982, in Cheltenham, at the Postgraduate Medical Centre.

Cases were demonstrated on Friday, 5th November, followed by dinner, which was also held in the Centre. At a Business Meeting the following were invited to join the Club:

Dr. A. Mourant (Truro) Cardiologist
Dr. J. Vann Jones (Bristol) Cardiologist
Dr. J. Gibson (Plymouth) Neurologist

It was agreed that the next meeting would be held in Plymouth on 29th and 30th April 1983.

The following papers were given and also Surgeon Commander J. G. Williams, RN, spoke on his experiences in the Falklands operation.

### CENTRAL NEUROFIBROMATOSIS

**S. Huson and David Thrush, Plymouth**

Six members in two generations of a family with central neurofibromatosis were reviewed, Three of the family had been diagnosed initially as having atypical, familial otosclerosis and the problems of early diagnosis were discussed.

In addition to bilateral acoustic neuromas, the hallmark of central neurofibromatosis, five of the six had other brain or spinal cord tumours. Three of the family have died from the effects of their neuromas or from surgery, one committed suicide shortly after the death of his mother because of increasing disability and two are still alive, though severely handicapped.

The largest family recorded with 85 affected members, was reviewed, the outcome was similar to the family presented in particular the poor prognosis following surgery.

All reviews have been retrospective and surgery has been undertaken when the acoustic neuromas were large. The importance of a prospective study was emphasised. Potentially affected members are now being followed regularly by the same doctor in the hope that regular clinical, radiological and audiological screening will detect acoustic neuromas at an early stage and with improved operative techniques the neuromas may be removed without damaging the facial nerve.

Recent work on nerve growth factor and its possible use as a genetic marker was discussed.

### A SIGMOIDOSCOPY SERVICE WITH OPEN ACCESS TO GENERAL PRACTITIONERS

**S. P. Wilkinson, Gloucestershire Royal Hospital**

Many district general hospitals now permit general practitioners open access for barium enema examinations. In this hospital the waiting time had increased to as much as 9 months. Yet basic medical teaching is such that a barium enema should not be performed without prior sigmoidoscopy. In order to both reduce this unacceptable waiting time and to provide a more orthodox service it was agreed that general practitioner requests for barium enema would not be accepted until a sigmoidoscopy had been carried out.

During the first 18 months 482 examinations using the rigid sigmoidoscope (and proctoscope where indicated) were carried out. In 146 (30%) an abnormality was found to account for the patients' symptoms. These included piles (73), inflammatory bowel diseases (36), malignant tumours (14), benign tumours (14), and others (9). A total of 97 of 146 general practitioners had used the service. Of the 97 using the service 92 replied to a questionnaire assessing its value. Ninety-one found it to be very useful or useful. Only one thought it unhelpful.

The waiting time for sigmoidoscopy remained less than two weeks and that for subsequent barium enema was reduced to less than one month.

### THE HEMIPLEGIC UPPER LIMB

**R. Langton Hewer, Bristol Royal Infirmary**

The poor prognosis for recovery of arm function in stroke patients is well known. However, few detailed studies have been undertaken.

Survivors of a stroke (56) were followed for between 2 and 3 years. A series of seven simple arm function tests were applied at intervals – as soon as possible after the stroke and then at 3, 6, 12 and 24 or 36 months. Those who scored 0 or 1 were considered non-functional, those scoring 2 to 6 – partially functional, and those scoring 7 were considered to be fully functional.

### RESULTS

1. In most patients – measurable recovery had ceased by 3 months post-stroke.

2. Eight (14%) of patients showed improvement beyond 3 months of whom five showed improvement beyond 6 months.

3. Twenty-six patients had an initial non-functional arm. Four (15%) eventually became fully functional and four partially functional.

4. The arm of one patient made an unexpectedly good recovery after the contra-lateral arm became useless.

Additional tests are now being used to increase the sensitivity of the assessment procedure; these include the finger tap rate and the ability to track a target with the affected fingers. We are now using a battery of tests to study patterns of recovery and to assess various types of therapy.

### AMAUROSIS FUGAX – MEDICAL OR SURGICAL TREATMENT?

**B. J. Kirby and C. R. Bayliss,**
**Royal Devon and Exeter Hospitals**

Transient monocular blindness (amaurosis fugax) is a distressing symptom. It can be made worse when the patient's doctor lulled into a false sense of security by the absence of physical signs, tries to be reassuring, as many of these patients will go on later to develop a stroke. This condition is a marker for the presence of carotid artery disease. A few cases are associated with haematological or cardiac disorders, but in the majority it is due to atheromatous disease of the cerebral circulation.

Twenty five patients presenting to the West of England Eye Infirmary since 1976 aged between 42 and 73 years had experienced a variable number of attacks affecting either the right (11), left (12) eyes, or both eyes on different occasions (2). A bilateral carotid bruit was present in six, an ipsilateral bruit in eight, a contralateral bruit in two and no bruit in nine. Arch aortograms were carried out in 12 and confirmed the presence of significant blockage in 85%, but usually the disease was extensive and involved the contralateral vessels too. Patients were started on antiplatelet aggregating agents and promptly lost the symptom or had a great reduction in its incidence. Two developed a stroke and two permanently lost the vision of an eye; one patient both lost vision in an eye and had a stroke; but these complications all arose when patients were not being treated. Only if surgery had a lower risk than medical treatment would operation be justified. Moreover the extent of the disease discovered clinically and shown on the arteriographic studies suggests that surgery on one vessel would be unlikely to halt the disease. Therefore we conclude that unless the disease is predominantly localised to one vessel drugs offer the best outlook for most patients.

